# Management of Metabolic-Associated Fatty Liver Disease/Metabolic Dysfunction-Associated Steatotic Liver Disease: From Medication Therapy to Nutritional Interventions

**DOI:** 10.3390/nu16142220

**Published:** 2024-07-11

**Authors:** Mohammad Beygi, Salma Ahi, Samaneh Zolghadri, Agata Stanek

**Affiliations:** 1 Department of Agricultural Biotechnology, College of Agriculture, Isfahan University of Technology (IUT), Isfahan 8415683111, Iran; m.beygi40@gmail.com; 2 Research Center for Noncommunicable Diseases, Jahrom University of Medical Sciences, Jahrom 7414846199, Iran; salmaahi.61@gmail.com; 3 Department of Biology, Jahrom Branch, Islamic Azad University, Jahrom 7414785318, Iran; 4 Department of Internal Medicine, Angiology and Physical Medicine, Faculty of Medical Sciences in Zabrze, Medical University of Silesia, Batorego 15 St., 41-902 Bytom, Poland

**Keywords:** fatty liver disease, nutrition, lifestyle, cardiovascular diseases, diabetes, obesity

## Abstract

Non-alcoholic fatty liver disease (NAFLD) is a common long-lasting liver disease that affects millions of people around the world. It is best identified with a hepatic fat build-up that ultimately leads to inflammation and damage. The classification and nomenclature of NAFLD have long been a controversial topic, until 2020 when a group of international experts recommended substituting NAFLD with MAFLD (metabolic dysfunction-associated FLD). MAFLD was then terminologically complemented in 2023 by altering it to MASLD, i.e., metabolic dysfunction-associated steatotic liver disease (MASLD). Both the MAFLD and the MASLD terminologies comprise the metabolic element of the disorder, as they offer diagnostic benchmarks that are embedded in the metabolic risk factors that underlie the disease. MASLD (as a multisystemic disease) provides a comprehensive definition that includes a larger population of patients who are at risk of liver morbidity and mortality, as well as adverse cardiovascular and diabetes outcomes. MASLD highlights metabolic risks in lean or normal weight individuals, a factor that has not been accentuated or discussed in previous guidelines. Novel antihyperglycemic agents, anti-hyperlipidemic drugs, lifestyle modifications, nutritional interventions, and exercise therapies have not been extensively studied in MAFLD and MASLD. Nutrition plays a vital role in managing both conditions, where centralizing on a diet rich in whole vegetables, fruits, foods, healthy fats, lean proteins, and specific nutrients (e.g., omega-3 fatty acids and fibers) can improve insulin resistance and reduce inflammation. Thus, it is essential to understand the role of nutrition in managing these conditions and to work with patients to develop an individualized plan for optimal health. This review discusses prevention strategies for NAFLD/MAFLD/MASLD management, with particular attention to nutrition and lifestyle correction.

## 1. Introduction

In a 1980 study, Ludwig et al. devised the first definition of liver disease independent of alcohol consumption. They identified a condition similar to alcoholic hepatitis in individuals with minimal alcohol intake, often accompanied by obesity or related diseases such as diabetes mellitus [1]. This condition was termed non-alcoholic steatohepatitis to describe the liver inflammation and damage seen in these patients. In 1986, non-alcoholic fatty liver disease (NAFLD) was coined by Shaffner and Thalerin [2]. NAFLD is a growing concern throughout the world and affects up to 38% of adults around the world. This chronic liver disease is particularly described by (i) metabolic dysfunction and (ii) systemic insulin resistance, which both contribute pathogenically to the incidence of liver morbidities such as cirrhosis, liver failure, and even hepatocellular carcinoma. Moreover, NAFLD is associated with extrahepatic complications, including cardiovascular disease (CVD), type 2 diabetes mellitus (T2DM), chronic kidney disease (CKD), and specific types of cancer [3,4,5].

In 2020, experts were inspired to evolve the terminology, transitioning from NAFLD to MAFLD. In general, NAFLD and NASH emphasize the histological aspects and differential diagnosis (i.e., non-alcoholic) instead of benefiting from a positive criterion. Instead of a negative connotation (i.e., nonalcoholic), MAFLD addresses a positive (i.e., metabolic-associated) diagnostic criterion. This groundbreaking change marks a departure from the only classification of patients according to hepatic steatosis (HS) without a clear cause, towards inclusion criteria that encompass metabolic dysfunction and accompanying risk factors. It signifies a deeper understanding of the condition and its underlying pathophysiology, bringing the diagnosis into line with the wider spectrum of metabolic disorders and their related health implications, such as insulin resistance, oxidative stress, and inflammation [6,7]. In June 2023, prominent multinational liver associations planned to replace the term NAFLD with MASLD based on a multisociety Delphi consensus to better reflect the multisystem nature of the disease [8]. However, there are still some issues with differentiating MASLD from its earlier designations (NAFLD and MAFLD) in terms of characteristics and mortality outcomes. MASLD is associated with an elevated risk of CVDs, making it a significant health concern. Many of the risk factors for CVDs are characteristic of MASLD (e.g., metabolic syndrome), which contributes strongly to comorbidities such as T2DM, hypertension, and CKD. Its prevalence is growing around the world due to changes in lifestyle and dietary habits. Early diagnosis and control of MASLD through lifestyle changes and medical interventions are crucial to preventing its complications and improving outcomes for affected individuals [9].

Importantly, shifting from one previously defined designation to a new one should reveal novel features to better differentiate characteristics, incidence rate, risk factors, underpinning mechanisms, burden, and mortality outcomes of this condition. Similarly, to devise a new terminology, its acceptance by the medical community and patients is of great importance. For example, the terms “fatty” and “nonalcoholic” may appear to be stigmatizing, and the terminology “steatotic liver” may be quite overarching (i.e., it may be felt to embrace a variety of etiologies for steatosis). Again, it could be crucial to furnish the terminology “MASLD” with some associated cardiometabolic risk factors. Therefore, the terminology for this disease should improve awareness and patient identification to help minimize its significant economic and clinical burden worldwide. It is critical to assess the health burden of this condition and predict its health care impact, as well as defining a dynamic terminology that can be upgraded with emerging data to allow for early diagnosis and devising tailored preventive measures. It is important to note that the introduction of MAFLD and MASLD does not replace the importance of lifestyle modifications as a primary treatment approach for NAFLD. These modifications include weight loss, dietary changes, and exercise. However, these new terminologies can help clinicians better diagnose, manage, and monitor NAFLD patients with underlying metabolic risk factors.

Generally, the emergence of MAFLD and MASLD represents a significant advancement in the understanding and management of fatty liver disease. These new designations offer a more nuanced and comprehensive framework for characterizing and managing the disease and assessing its impact on patient outcomes. By embracing these new terminologies and elucidating their associated implications, the medical community can enhance awareness, identification, and management of NAFLD, paving the way for more effective interventions and improved patient care. This review aims to offer various therapy plans for controlling and overcoming NAFLD, spanning from medication therapy to nutritional interventions. In addition, this review is assumed to provide new insight about this condition that can be useful for both patients and therapists.

## 2. MAFLD/MASLD Management

**Figure 1:**
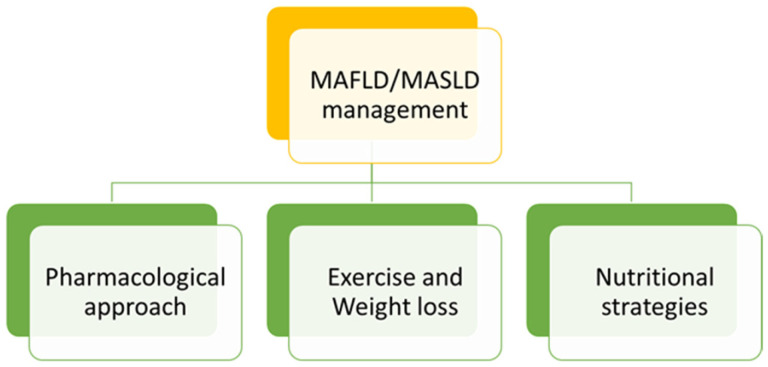
Therapy plans for MAFLD/MASLD management.

As stated, MAFLD/MASLD treatment, beyond focusing on liver dysfunction, requires a holistic and multidisciplinary approach to manage metabolic syndrome manifestations (e.g., hypertension, dyslipidemia, obesity, and T2DM). Accentuating the centrality of metabolic dysfunction and measuring the features of metabolic syndrome are essential in this approach. When treating patients with MASLD and CVD, it is crucial to consider a treatment plan addressing both conditions. Importantly, there exists a tight nexus between CVDs and NAFLD/MASLD that is controlled by key pathophysiological pathways. NAFLD is supposed to correlate with cerebral, peripheral vascular, and coronary events, as well as with preclinical atherosclerotic injury. Pathophysiologically, NAFLD links with insulin resistance (and thereby interrupted production of adipokine, and particularly adiponectin). Likewise, excess production of ROS (reactive oxygen species) inflicts elevated oxidative stress, culminating in oxidized free fatty acids [8], de novo lipogenesis (DNL), and overaccumulation of triglycerides. More specifically, such a nexus is controlled by a multitude of factors, including (1) endothelial dysfunction, (2) elevation in the rates of TNF-α, IL-6, MCP-1 (monocyte chemoattractant protein-1), and C-reactive protein (CRP), (3) enhanced levels of metalloproteinases in extracellular vesicles (EVs), (4) triggered asymmetric dimethylarginine, and (5) decreased levels of adipokine (particularly, adiponectin). At the same time, cardiometabolic risk factors are strongly in play. These include oxidative stress, lipid metabolism, systemic inflammation, and dysfunction of adipose tissues. Collectively, all of these result in cerebrovascular and peripheral arterial diseases, elevated risk of arrhythmias, mass and diastolic dysfunction, and remodeling of the heart’s left ventricle [10]. A treatment that can improve CVD risk factors (i.e., hypertension, atherogenic dyslipidemia, dysglycemia, and abdominal obesity) in MASLD patients would also, ideally, have positive results in HS, inflammation, and fibrosis. T2DM not only multiplies the risk of both microvascular (MVD) and macrovascular disease (MAVD), but it further can uplift the risk of CKD, which itself can elevate the risk of CVDs [11]. It is crucial to manage hyperglycemia using glucose-lowering agents to attenuate the risk of MVD and lessen the risk of renal end-organ damage that will ultimately alter the risk of CVDs. In addition to medication therapies that prevent CVD, healthcare professionals should advocate for healthy lifestyle changes in individuals with MASLD. In the following, this review mentions various therapy plans for MAFLD/MASLD management (Figure 1).

### 2.1. Pharmacological Approach

Despite the paucity of placebo randomized controlled trials (RCTs) to test the effect of medications on CVD outcomes, particularly among MASLD patients, researchers benefit from endpoint justifications in cardiovascular trials and then extrapolate this evidence to MASLD patients. Reflecting on all aspects of medications (i.e., benefits, side effects, and licensed indications), researchers explore if these drugs can synergistically attenuate the risks of CVDs and alleviate MASLD. It is crucial to address both conditions to ensure that patients receive comprehensive care and achieve optimal health outcomes. Consequently, researchers have reflected on and organized all aspects of drugs, including their grouping, primary status and site of functioning, administration tips, advantages, and adverse effects, which all can potentially reduce the risk of CVDs in patients with MASLD. For patients suffering from obesity, agonists of the incretin receptor (IR) can induce ~10% to 15% reduction in body weight [12]. Researchers have also summarized a pragmatic approach to the assessment and management of CVD risk in adults with MASLD. It is important to note that finding a suitable treatment for MASLD patients with CVD can be challenging. However, considering drug actions that improve cardiometabolic risk factors while remaining neutral or potentially beneficial for liver disease in MASLD, patients can receive the best possible treatment for their condition. For example, Targher et al. (2024) suggested a combination therapy approach targeting the liver and attenuating elevated risks of CVD or treating both obesity and T2DM. They reported the potential use of IR agonists, SGLT2 inhibitors, statins, and inhibitors of the renin-angiotensin (RA) system. They also recommended pioglitazone to manage NASH/MASH in some patients [13]. In the following, some medication therapies are summarized.

Antihyperglycemics: Compared to the former antihyperglycemic agents, both “GLP-1 agonists” and “SGLT2 inhibitors” are well known for their cardiovascular and weight-modifying benefits. The GLP-1 antagonists work by imitating the action of natural GLP-1, which promotes insulin secretion, hampers glucagon secretion, decelerates gastric emptying, and promotes satiety. These agents have shown significant cardiovascular benefits, including a decrease in the risk of MACE (major adverse cardiovascular events) (e.g., cardiac and cerebral attacks and cardiovascular mortality in T2DM patients) [14]. Similarly, SGLT2 inhibitors block renal glucose reabsorption pathways, culminating in elevated urinary glucose excretion. Beyond their glucose-lowering effects, SGLT2 inhibitors have demonstrated cardiovascular benefits, including reductions in hospitalization of T2DM patients with cardiovascular events and heart failure. Additionally, SGLT2 inhibitors have been associated with weight loss, likely due to calorie loss through increased urinary glucose excretion [15]. The cardiovascular and weight-modifying benefits of GLP-1 agonists and SGLT2 inhibitors represent significant advancements in the therapy of T2DM, providing patients with additional therapeutic options beyond traditional antihyperglycemic agents. The efficacy of new forms of antihyperglycemics on HS indices (i.e., FLI = fatty liver index and HIS = hepatic steatosis index) has been studied. As reported from a 26-week study on 174 HS patients with obesity and T2DM, the combined use of “SGLT2is + GLP-1RAs” provides more measurable results, compared to single drugs, in terms of glucose control, weight loss, reduction in waist and abdominal circumference, and drop in HS biomarkers [10]. A similar meta-analysis revealed the marked contribution of SGLT2Is to reduction in hepatic enzymes and fat and improvement in body composition in NAFLD patients with T2DM. As reported, SGLT2Is outperform other antihyperglycemics in reducing HbA1c (glycosylated hemoglobin A1c) and FPG (fasting plasma glucose). However, the efficacy of SGLT2Is in reducing HOMA-IR (homeostatic model assessment of insulin resistance) (i.e., improving insulin resistance) is lower than that of other antihyperglycemics. Similarly, SGLT2Is outperform other antihyperglycemics in reducing levels of ALT (alanine transaminase), AST (aspartate transaminase), and FIB-4 (fibrosis-4 index for liver fibrosis). Ultimately, SGLT2Is are superior to other antihyperglycemics in reducing body weight, PDFF (proton density fat fraction), VFA (visceral fat area), and subcutaneous fat areas [16].

GLP1 Agonists: Indeed, numerous studies have demonstrated the potential benefits of GLP-1 receptor agonists in NAFLD, particularly in those with diabetes. GLP-1RAs have been associated with improvements in serum transaminases, weight loss, a marked reduction in HS, and a general improvement in non-alcoholic steatohepatitis [17]. However, the findings on the impact of GLP-1RAs on hepatic fibrosis are somewhat contradictory, and some studies indicate a potential reduction in fibrosis [18]. While there is promising evidence of the positive effects of GLP-1RAs on various aspects of NAFLD, more research is needed to fully understand their impact on liver fibrosis and to address any inconsistencies in the current body of evidence. Nonetheless, the overall potential of GLP-1RAs in managing NAFLD, especially in individuals with diabetes, is evident from existing research. In a randomized double-blind phase 2 trial in four UK medical centers, subcutaneous liraglutide (1.8 mg daily) demonstrated efficacy in resolving definite non-alcoholic steatohepatitis [17] without worsening of the fibrosis compared to placebo over 48 weeks, and 39% of liraglutide-treated patients achieved resolution versus 9% in the placebo group. Adverse events, mainly gastrointestinal, were mostly mild to moderate [19].

SGLT2 Inhibitors: A five-year retrospective study assessed the outcomes of long-term SGLT2 inhibition therapy (specifically canagliflozin) in patients with T2DM and NAFLD. Liver biopsies obtained at pretreatment and 24 weeks, 3 years, and 5 years post-treatment revealed that 50% of patients experienced histological improvement, with notable reductions in steatosis, inflammation, and fibrosis stage, indicating a favorable impact of SGLT2 inhibition therapy on NAFLD in T2DM patients over 5 years [20]. An analysis of five-year RCT showed that treatment with SGLT2 inhibitors (SGLT2Is) led to a measurable decrease in liver fat content and an increase in the liver-to-spleen ratio in various studies. Additionally, alanine aminotransferase [21] levels consistently decreased with SGLT2I treatment. Notably, dapagliflozin treatment resulted in a significant decrease in liver stiffness measurements among patients with significant fibrosis [22]. In another meta-analysis conducted in July 2020, which included 10 articles with 555 patients from seven RCTs and three cohort studies, significant improvements were observed in various parameters after SGLT2I treatment compared to controls. These improvements included hepatic fat content, AST, ALT, body composition, glycemic control, lipid parameters, and inflammatory markers [23]. A multicenter retrospective study explored the long-term effects of SGLT2Is in patients with NAFLD and T2DM. Among the 1262 patients with T2DM receiving SGLT2Is, 202 with NAFLD were analyzed over 48 weeks. Significant reductions in body weight, liver enzymes, plasma sugar content, hemoglobin A1c, and FIB-4 (Fibrosis-4) index were observed at 48 weeks, with the median FIB-4 index decreasing from 1.42 to 1.25. Notably, significant reductions persisted over 3 years of administrating SGLT2Is, especially in intermediate- and high-risk FIB-4 index groups, indicating a favorable antifibrotic effect of SGLT2Is in NAFLD patients with T2DM [24]. Although numerous human studies have highlighted the beneficial effects of SGLT2Is in the treatment of NAFLD, particularly in patients with T2DM [25], there is limited research on NAFLD in non-diabetic patients. There is only a single-center 121-week study that enrolled 22 patients, 12 receiving dapagliflozin and 10 receiving teneligliptin, a DPP4 inhibitor. This research reported decreases in serum transaminases in both treatment groups, with additional reductions in total body fat and water content, leading to a decrease in total body weight specifically in the dapagliflozin group [26].

Thiazolidinedione (TZD): TZDs, including pioglitazone and rosiglitazone, are known as agonists of PPARγ. PPARγ, expressed across various tissues, regulates energy balance, lipid storage, and intra-abdominal and subcutaneous adipose tissue redistribution by facilitating triglyceride buildup in peripheral fat cells [27,28]. TZDs, particularly pioglitazone, decrease free fatty acid levels by promoting adipogenesis, aiding in fatty acid storage within adipose tissue. Additionally, TZDs improve insulin sensitivity in the liver, skeletal muscles, and adipose tissue. This increased sensitivity enhances glucose uptake by peripheral tissues and the liver, improving glucose utilization and decreasing hepatic glucose output [29].

Despite the benefits of these interventions on CVD events, more research is necessary to determine the long-term effects. Furthermore, though medication may manage NAFLD/MAFLD/MASLD with advanced liver fibrosis or other complications, lifestyle modifications are the cornerstone of treatment, and medication should be helpful in conjunction with these interventions [30,31]. Importantly, the extremely complex pathophysiology of this condition requires a pharmacological intervention that can target multiple sites that account for the disease (i.e., adipose tissue, the cardiovascular system, the gut). Likewise, there is a need for a tailored therapy plan for each patient. For example, those patients suffering from osteoarticular issues are less likely to adopt lifestyle modifications (particularly, physical activity for weight loss) to substantially reduce fat mass. Similarly, some genetic factors may hinder or interrupt regular response to weight loss (as the major therapy plan), particularly in diabetic patients, and multiply the chance of NASH occurrence. Thus, the therapy plan for these patients shall be based more on pharmacological interventions than on lifestyle modifications. In addition, the safety and efficacy of drugs for all diverse patient groups need to be approved within clinical trials. For example, the long-term effect of medications on vital indications (e.g., body weight, blood pressure, profile of lipids, glycemic control, renal function) should be critically explored, particularly in CVD patients [32,33].

### 2.2. Exercise and Weight Loss

One of the primary risk factors for developing NAFLD, MASLD, and MAFLD is obesity. Excess weight, especially around the waistline, is strongly associated with the accumulation of fat in the liver, leading to inflammation and potential scarring. As a result, addressing weight loss is a critical component of managing and potentially reversing the progression of these conditions [34]. Weight loss plays a key role in improving liver health for individuals with NAFLD, MAFLD, and MASLD in several important ways. Firstly, shedding excess pounds can lead to a reduction in liver fat accumulation. Even a modest weight loss of 5–10% has been shown to significantly decrease liver fat content, which can help alleviate inflammation and prevent further liver damage. In addition to reducing liver fat, weight loss is beneficial for addressing the systemic metabolic dysregulation often present in individuals with NAFLD, MASLD, and MAFLD. Losing weight can improve insulin sensitivity, reduce blood sugar levels, and lower the levels of fats in the bloodstream, all of which are vital for managing metabolic health and reducing the risk of complications such as T2DM and CVD. Furthermore, weight loss has been shown to improve liver enzyme levels, which are often elevated in individuals with fatty liver disease. Lowering these enzymes through weight loss signifies a reduction in liver inflammation and can indicate an improvement in overall liver function. It is important to note that while weight loss is crucial for managing NAFLD, MASLD, and MAFLD, how it is achieved matters as well. Sustained, gradual weight loss through a combination of dietary modifications, increased physical activity, and behavior change is generally recommended for individuals with fatty liver disease. Crash diets and rapid weight loss can potentially exacerbate liver damage and are not advisable for these individuals [35,36]. Generally, the importance of weight loss in managing NAFLD, MASLD, and MAFLD cannot be overstated. Addressing excess body weight can lead to a reduction in liver fat, improved metabolic health, and a decreased risk of disease progression and complications. As such, individuals diagnosed with these conditions should work closely with healthcare professionals to develop personalized weight loss strategies that prioritize both liver health and overall well-being.

#### 2.2.1. Exercise

For individuals diagnosed with MAFLD/MASLD, incorporating regular exercise into their routine can be highly beneficial. The different types of exercise can play a crucial role in managing these conditions and improving overall health. Here, we will explore the various types of exercises that can be particularly beneficial for individuals with MAFLD/MASLD [37].

##### Aerobic Exercises

Aerobic exercises, also known as cardiovascular exercises, are great for individuals with MAFLD/MASLD. Activities like walking, jogging, cycling, swimming, and aerobic dance can be excellent choices to help improve cardiovascular health and are effective in burning calories, which can aid in weight management. It is recommended that individuals aim for at least 150 min of moderate-intensity aerobic activity each week [38].

##### Strength Training Exercises

This type of exercise can improve muscle strength and increase metabolism and is particularly beneficial for individuals with MAFLD/MASLD, as it can aid in weight management and improve insulin sensitivity. Additionally, strength training can help prevent the loss of muscle mass, which is important for overall health and mobility. Incorporating activities such as lifting weights, resistance band exercises, and bodyweight exercises into a workout regimen can be highly beneficial [37].

##### Flexibility and Balance Exercises

Activities such as stretching exercises, tai chi, and yoga can help improve flexibility, balance, and overall physical well-being [38]. These training activities are particularly important for individuals with MAFLD/MASLD, as they can help maintain mobility and reduce the risk of injury.

##### High-Intensity Interval Training (HIIT)

HIIT involves alternating between short bursts of intense exercise and periods of rest or lower-intensity exercise. It can be an effective approach to increase metabolism and improve cardiovascular health for individuals with MAFLD/MASLD [39].

Generally, incorporating aerobic exercise, strength training, flexibility and balance exercises, and high-intensity interval training can have significant benefits for individuals with MAFLD/MASLD. Regular physical activity plays a key role in managing these conditions and improving overall health. By finding the right balance and consulting with healthcare professionals, individuals can develop an exercise routine that supports their health and well-being in the context of MAFLD/MASLD.

### 2.3. Nutritional Strategies

Maintaining a healthy weight and improving metabolic syndrome features are vital to prevent MAFLD/MASLD complications and control disease progression. Therefore, disease management will be fulfilled by focusing on lifestyle changes, including exercise, quitting smoking, dietary changes, and calorie restriction, to reduce liver fat content and improve liver function. Alcohol abstinence should be recommended to benefit liver health, and healthcare professionals need to consider the benefits and uncertainties of weight loss and calorie restriction in MASLD patients. Thus, avoiding excessive alcohol consumption is crucial, as alcohol can worsen liver inflammation and damage. While there is an ongoing dispute on whether controlled daily consumption of alcohol is beneficial or harmful, the consumption of alcohol has increased among people and therefore can frequently coexist with metabolic syndrome. Research advocates that a mild-moderate intake of alcohol is associated with an elevated risk of liver outcomes. Therefore, healthcare professionals need to ban alcohol consumption for those with MASLD to benefit from optimal liver functioning [40].

Maintaining a healthy lifestyle is crucial for individuals with MASLD to prevent CVDs. The diet plays a pivotal role in the management of NAFLD/MAFLD/MALD. Healthcare professionals should advise patients to quit smoking and, particularly, adopt a healthy diet that is low in saturated and trans fats and refined carbs and, instead, rich in fruits, whole grains, vegetables, and lean proteins [31]. The role of vegetable sources of protein should not be overlooked. By incorporating various plant-based protein sources into their diet, individuals can benefit from essential nutrients, fiber, and a potentially reduced intake of unhealthy fats [41]. These food choices should be combined with fish or lean animal protein and avoiding the consumption of excess trans fats, processed and red meats, refined carbs, sweetened drinks, sucrose, and fructose. When suggesting the benefits of lifestyle modifications to specifically fight obesity and alleviate CVD risk in patients with MASLD, it is essential to know that maximum weight loss occurs with calorie restriction. For overweight or obese individuals, calorie restriction and weight loss are beneficial for reducing liver fat and inflammation, improving metabolic syndrome characteristics [42,43,44], and managing NAFLD/MAFLD/MALD [45]. Even a modest weight loss of 5 to 10% can significantly improve liver health and reduce the risk of disease progression. Weight loss can occur through dietary adjustments and regular physical activities [46]. Exercise helps reduce liver fat and improve insulin sensitivity. It is recommended to engage in minimally 150 min of moderate aerobic exercise weekly, in conjunction with strength training (two sessions, weekly) or 75 min of vigorous physical activity each week [47]. This helps maintain a healthy weight and prevent CVD. A diabetes remission clinical trial (DiRECT) has revealed that marked calorie restriction induces T2DM remission in overweight or obese individuals recently identified with T2DM. According to this trial, burning body fat is essential to reduce glucose concentration in plasma, alleviate metabolic syndrome, and ameliorate MASLD. However, the sustainability of these benefits is unclear. It is also ambiguous whether improving cardiometabolic risk factors on calorie restriction and weight loss can result in long-lasting benefits for CVDs [13].

Regular monitoring and follow-up are essential in the management of NAFLD/MAFLD/MASLD. This includes regular liver function tests, imaging studies to assess liver fat content, and screening for complications such as liver fibrosis and hepatocellular carcinoma. It is important to work carefully with healthcare professionals to implement a tailored management plan and address any underlying metabolic disorders.

Today, lifestyle modalities such as dietary modifications and bodily motions (e.g., to avoid obesity) are the subject of debate. For example, although burning body fats is claimed to reduce intrahepatocellular lipid (IHL) levels, there is a dearth of research elucidating how factors such as nutritional delivery (i.e., macro- and micronutrients) and dietary habits may be promising [48]. Likewise, while Mediterranean diets can potentially reduce the disease without the need to lose weight, other stimuli, such as prolonged fructose-enriched regimes and the adoption of a sedentary lifestyle, are supposed to worsen HS [49]. Clinical progress is correspondingly trivial with respect to pharmaceutical therapies (particularly NASH, a key contributor to hepatocellular carcinoma, HCC), while managing metabolic causes of this condition (e.g., by targeting miRNAs, GLP-1, PPARs) is in the early stages [50]. In addition, it should be noted that most studies so far have explored NAFLD, whereas MAFLD and MASLD are novel concepts that differ from the former in several characteristics. For example, research reveals that MAFLD is characterized mostly by advanced age, T2DM, HOMA-IR values exceeding optimal cut-off limits, hypertension, higher BMI, and elevated hepatic enzymes [51], and it better diagnoses patients with fibrosis and HS than its conventional NAFLD terminology [52]. Indeed, when accompanied by metabolic disorders, the therapy of HS requires new criteria to be considered, such as metabolic dysregulation (i.e., CSSI (elevated inflammatory cytokines), hepatic insulin resistance (HIR), IFG (impaired fasting glycemia), hypertriglyceridemia, hypertension, etc.), T2DM, and obesity [53]. As mentioned above, patients with both MASLD and MAFLD exhibit a worse metabolic profile, compared to those with only MASLD. Consequently, due to limited research on the management of MASLD as a new term, this section explores the main therapy options for MAFLD, including nutritional strategies (by adopting a balanced diet and promoting weight loss and physical activities), addressing the underpinning metabolic disorders, and therapeutic interventions.

#### 2.3.1. Dietary Interventions for MAFLD

Dietary interventions [54] for MAFLD are based principally on a balanced diet and the adoption of a lifestyle that concomitantly maximizes energy expenditure, restricts energy consumption, and promotes physical activities [21]. For example, while gradual weight loss (by 7 to 10%) in conjunction with the adoption of a Mediterranean diet (MD) (i.e., for the intake of more vegetables and fruits, cereals and pseudocereals, high-fiber foods and unsaturated fatty acids (FAs)) is effective in alleviating MAFLD, parallel indulgence in unhealthy meals (e.g., saturated FAs, processed foods, refined sugar- or starch-based CHOs) causes success to go down the tubes [41,55]. Or, while aerobic exercise, resistance exercise, and low-carb regimes all improve hepatic fat content (HFC), triglyceride (TG), glycosylated hemoglobin A1c (HbA1c), high-density lipoprotein (HDL), and homeostatic model assessment for insulin resistance (HOMA-IR) levels (particularly in obese patients with MAFLD), “a diet low in carbs” is better than “a diet low in fats” in improving HFC levels, and “resistance exercise” outperforms “aerobic exercise” in improving HFC and TG levels [56]. Furthermore, several studies have indicated regular coffee consumption may be associated with a reduced risk of developing MAFLD and its progressive stages due to the impact of coffee on liver enzymes such as alanine aminotransferase [21] and aspartate aminotransferase (AST). Studies have suggested that regular coffee consumption may help in lowering the levels of these enzymes, indicating a potential protective effect on liver health. Moreover, the bioactive compounds present in coffee, such as chlorogenic acid and caffeine, have been proposed to exert antioxidant, anti-inflammatory, and metabolic regulatory effects that could contribute to the observed benefits on liver enzymes and overall liver health. These bioactive compounds may play a role in mitigating liver damage and inflammation, thus potentially slowing the progression of MAFLD. However, it is important to note that the relationship between coffee consumption and MAFLD is complex and multifactorial. Factors such as the type of coffee, brewing method, and individual variations in response to coffee intake can influence its impact on liver health. Additionally, excessive consumption of highly sweetened or fatty coffee beverages may counteract any potential benefits and could contribute to metabolic disturbances and liver damage. As a result, a balanced diet tailored to each patient with characteristic features is a prerequisite for successful DI.

##### Diet Low in Carbohydrates

When a threshold is exceeded for the body’s need for energy, the process of DNL converts surplus carbohydrates into triglycerides, thus provoking HS in those overriding high-carb diets. The same is the case for free sugars (notably fructose), as high-fructose diets make the person predisposed to HS by activating DNL during its metabolism [57]. For example, Schwimmer et al. enrolled male youths (aged 11–16) in a follow-up for 8 weeks and found a noticeable drop (i.e., from 25% to 17%) in HS in groups receiving “free-sugar-low diets” rather than a trivial drop (i.e., from 21% to 20%) in those receiving normal diets, suggesting a key role that free sugars may play in the progression of this disease [58]. In addition to the direct contribution of carbohydrates to MAFLD reported in the literature, these macronutrients can also be indirectly linked to the onset and progression of the disease. The relationship between carbohydrates, diet drinks, and MAFLD is a complex interplay that demands greater attention and research. Wu et al. reported that overuse of diet drinks (even claimed to be zero in sugar and calories) can trigger the appearance of MAFLD through a mediating role of BMI. They studied 2378 participants (1089 with MAFLD) and found that excessive use of diet drinks results in BMI-mediated occurrence of this disease, with BMI mediating this nexus in 87.7% of cases [59].

These findings underscore the indirect impact of carbohydrates in the form of diet drinks on MAFLD, and suggest that the definition of optimal levels of carbohydrates in each diet should reflect multiple interrelated factors such as age, underlying diseases, sex, dietary habits and customs. Table 1 summarizes findings of some studies exploring the role of carbohydrates in MAFLD.

As presented in Table 1, research strongly advocates that carbohydrate-rich diets contribute a great deal to the incidence and progression of HS. Therefore, recent studies have explored the effect of low carbohydrate intake on the prevention of the disease. Table 2 presents recent findings on the therapy plans proposed in the literature to treat HS.

According to Table 2, the adoption of a low-carb diet is the first-line therapy for HS with respect to the contribution of carbohydrates. However, most of these findings are from studies targeting NAFLD, while current MAFLD terminology emphasizes exploring the disease along with other conditions, particularly CVDs and T2DM. That is, the metabolic aspect of MAFLD is strongly correlated with T2DM, and T2DM itself is tightly linked to CVDs [72]. Therefore, the therapy plan needs to be multifaceted and highly versatile to take into account numerous factors in play. Most importantly, more than 70% of patients with T2DM are affected by MAFLD (with advanced hepatic fibrosis that is observed in 20% of these patients), and T2DM patients are more susceptible to cirrhosis, hepatic failure, and HCC (compared to healthy individuals) [73]. This implies a strong and bidirectional correlation between MAFLD and T2DM, thus requiring a therapy plan that simultaneously targets both T2DM (e.g., by using pioglitazone or GLP1-RA) and risk factors for MAFLD. Research indicates that a reduction in glucose levels can reduce liver fat content. For example, when assessing data from 637 T2DM patients, it was found that glucose control (i.e., reduction in glycated hemoglobin) reduces the fatty liver index (FLI) in patients with co-existing T2DM and MAFLD, independent of weight loss [74]. However, as stated earlier, MAFLD is a broader concept that requires more reflection when studying HS. For example, in a study of data extracted from the US NHANES, it was found that of 6727 T2DM patients, 4982 had MAFLD (i.e., 2950 were NAFLD(+)/MAFLD(+), and 2032 were NAFLD(−)/MAFLD(+)), implying the potency of the new terminology in diagnosing HS, as it reflects more aspects that are exceedingly interconnected. Likewise, patients with NAFLD(−)/MAFLD(+) were found to be more susceptible to CVDs, advanced fibrosis, and CVDs and/or all-cause-based mortality, and these groups of patients were mostly older, females, and those with significantly higher BMI, suggesting a contribution of multiple factors to the development of HS in T2DM patients [75]. Elsewhere, research targeting patients from Turkey, Japan, and the USA (*n*: 2016; mean age: 57.8 years; mean BM: 31.3 kg/m^2^) revealed that NAFLD patients with T2DM are at increased risks of HCC and hepatic decomposition (i.e., over a follow-up of 1, 3, and 5 years) [76]. Furthermore, T2DM patients with NAFLD (particularly lean or non-obese NAFLD) have been reported to have higher risks of developing various cancers (e.g., cervical, breast, rectal, thyroid, bladder, and gastric cancer) [77].

HS is also associated with CVD, and emerging evidence suggests a strong link between NAFLD and CVDs such as arterial hypertension, coronary artery disease (CAD), and structural heart disease [78]. However, the new definition of MAFLD is reported to identify more patients with metabolically intricate HS and high risks of CVDs, as it reflects other CVD-contributing factors such as waist circumference (WC) (≥80 in females and ≥90 in males), hypertension (≥130/85 mm Hg), TG (≥150 mg/dL), and HDLC levels (<50 mg/dL in females and <40 mg/dL in males) [79]. For example, patients with MAFLD (compared to NAFLD ones) have been reported to have a higher 10-year CVD risk [80]. Similarly, a national cohort study in South Korea in 47–86-year-old participants revealed that compared to those without HS, patients with MAFLD, alcohol-related liver disease (ALD), and MetALD (MAFLD+ALD) are more likely to develop CVDs, and MetALD showed a higher contribution to CVDs than MAFLD alone [81]. With respect to the new terminology of the disease, HS will therefore be a daunting and intricate condition to overcome. Thus, instead of simple navigation on low-carbohydrate diets, it requires multifunctional and versatile therapeutic plans that cover multiple facets, similar to those currently being subjected to clinical trials for other diseases, particularly cancers and CNS diseases. For example, an Impact of anti-hyperglyceMic Agents on NAFLD proGressIoN in type 2 Diabetes (IMAGIN) study on 63 T2DM patients shows that those patients receiving combined “metformin + empagliflozin” therapy (compared to metformin alone) possess lower BMI (body mass index), glycated hemoglobin, ALT (alanine transaminase), CAP (controlled attenuation parameter), and steatosis degree [33]. Elsewhere, in a randomized, double-blind, placebo-controlled clinical trial, it was found that a 24-week therapy plan based on empagliflozin (compared to pioglitazone) significantly improved HS and fibrosis in NAFLD patients suffering from T2DM, while empagliflozin synergistically reduced body weight and burned abdominal fat in this group [82]. Similarly, a 24-week RCT explored the additive effect of ipragliflozin on “NAFLD + T2DM” patients receiving pioglitazone and metformin. In this study, those patients receiving ipragliflozin exhibited reduced hepatic fat content and whole-body visceral fat, suggestive of ipragliflozin efficacy in ameliorating HS and burning fat in “euglycemic T2DM + NAFLD” patients consuming metformin and pioglitazone [83].

**Figure 2:**
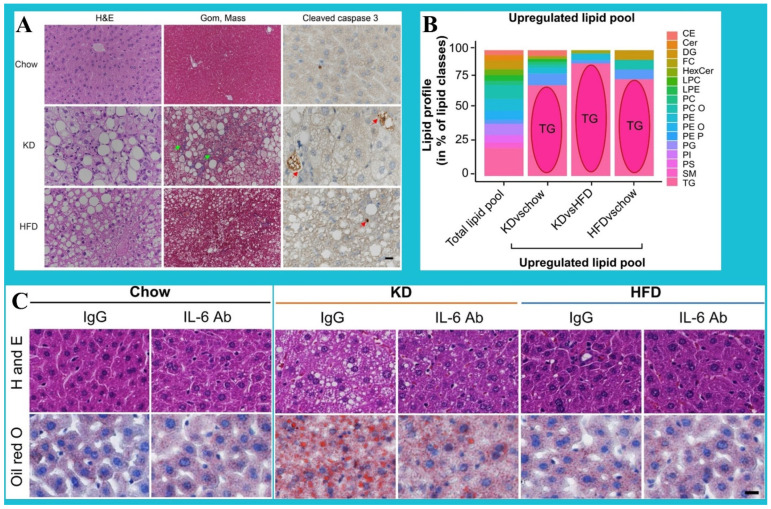
Effects of KD (i.e., consuming more fat and protein instead of carbohydrates) on the incidence and progression of hepatic conditions, compared to HFD and Chow. (**A**) Images taken from the liver sections (with scale bar = 100 μm), which are stained with H&D, cleaved caspase 3, and Gom and mass (for fibrosis). As shown, marked hepatic inflammation, ballooning of hepatocytes, and fibrosis are visible in mice fed with KD, compared to other treatments. Further, apoptotic hepatocytes can be found in KD-fed mice, suggestive of “caspase 3” activation in hepatocytes. (**B**) Lipid profile (i.e., distribution of lipid species in the pool of upregulated lipids, compared to the total pool). Under various treatments (i.e., KD vs. HFD vs. Chow), it can be seen that triglycerides (TGs) are overrepresented (compared to other species), suggestive of marked effect of KD on TGs. (**C**) Liver sections (stained with oil red O or H&S) (scale bar = 100 μm). As depicted, KD can potentially trigger IL-6 and JNK signaling pathway, which both are linked with glucose intolerance and HS. (KD: ketogenic diet; HFD: high-fat diet, IgG: immunoglobulin G; IL-6Ab: interleukin 6 antibody) [84]. This content is distributed under the terms of the Creative Commons Attribution 4.0 International license.

The next factor to consider is that a low-carb diet can be a double-edged sword, as following this dietary pattern may negatively trigger HS. For example, while a ketogenic diet (KD) is supposed to be beneficial for weight loss, it may trigger HIR, HS (particularly NASH), and fibrosis in C57BL/6 mice in vivo. Long et al. studied C57BL/6 mice and found that mice on KD with thermoneutrality are more prone to severe liver damage, inflammation, and steatosis than mice fed with HFD (high-fat diet). Likewise, KD was found to increase p-JNK signaling and IL-6 signaling rates, thus provoking HIR and steatosis. As illustrated in Figure 2A, severe steatosis and markedly elevated liver inflammation, fibrosis, ballooning, and NASH were more visible in KD-fed mice than in those receiving HFD and Chow diets. Furthermore, over-activated caspase3 in hepatocytes was demonstrated, suggestive of apoptotic hepatocytes in mice receiving KD. KD further increased the concentration of 50 and 41 various lipids (respectively, compared to Chow and HFD), with TG that was found to be selectively overexpressed in mice receiving KD (Figure 2B). Additionally, Figure 2C shows that KD induced the IL-6 and p-JNK signaling pathways that are associated with glucose intolerance and HS, while both neutralization of IL-6 and inhibition of the p-JNK pathway were effective in reversing KD-provoked glucose intolerance and alleviating HIR [84].

**Figure 3:**
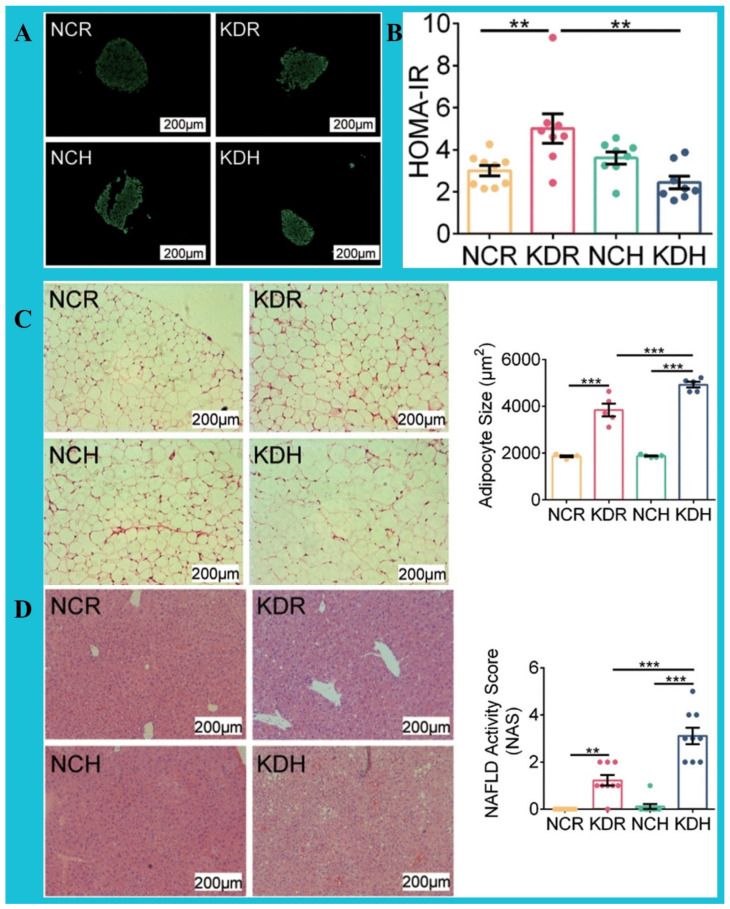
Effects of KDR (ketogenic diet; with 89.5% F, 0.1% C, 10.4% P), NCR (fed control normal chow of KDR; with 10% F, 70% C, 20% P), KDH (ketogenic diet’ with 91.3% 1% C, 7.7% P), and NCH (fed control normal chow of KDH; with 15.5% F, 64.5% C, and 20% P) diets on hepatic parameters in male C57BL/6J mice. (**A**,**B**) KDR triggers insulin resistance and impaired glucose homeostasis, more severely than other diets. (**C**,**D**) Hematoxylin and eosin (H&S) staining of the liver and epididymal adipose tissue (eAT) section and adipocyte area, implying higher scores of NAFLD and adipocyte size in mice fed with KDR and KDH (than NCH and NCR); these scores are higher in KDH than in KDR. F: Fat; C: Carbohydrates; P: Protein. (** *p* < 0.01; *** *p* < 0.001) [86]. This content is distributed under the terms of the Creative Commons Attribution 4.0 International license.

Thus, despite the fact that KD is considered metabolically beneficial (particularly for weight loss), some inconsistent reports imply its selective contribution to liver insulin sensitivity and glucose intolerance. For example, Grandl et al. reported that C57BL/6 mice fed with KD and HFD for 3 days appeared to be glucose-intolerant, with such intolerance associated with elevated lipid oxidation and decreased respiratory exchange ratio (RER) and more severe in KD-fed mice than in HFD-fed animals. They attributed this glucose intolerance to the dampened suppression of hepatic glucose production [85]. Similarly, Yue et al. found that KD formulations “KDR” (with 89.5% fat) and “KDH” (with 91.3% fat) both trigger impaired glucose and lipid metabolism in mice, with KDH provoking more fat accumulation in C57BL/6J male mice than KDR. Furthermore, KDR caused insulin resistance and impaired glucose homeostasis, with higher values of the HOMA-IR index and the insulin resistance index (Figure 3A,B). In addition, it was found that KDH results in higher scores for NAFLD and adipocyte size than other dietary groups (Figure 3C,D). Importantly, they linked these KD-triggered metabolic disorders to the source and volume of fat in the diet and assumed all of them to be probably due to alterations in the gut microbiota and metabolites [86]. Although these findings are from in vivo research on animal models, similar studies are worth exploring within human clinical trials to explore the exact impacts of various DIs on low-carb diets. Thus, a versatile therapeutic plan for these conditions needs to be multifaceted, reflect various factors, and be designed to cover multiple comorbidities in MAFLD.

As stated earlier, MAFLD is associated with metabolic conditions such as hypertriglyceridemia, elevated WC, arterial hypertension, low levels of HDL cholesterol, HIR, and prediabetes, while factors such as age can also be in play. For example, Theofilis et al. found that MAFLD correlates with cardiorenal risk markers in patients with “newly diagnosed hypertension”, with uric acid (cutoff ≥ 5.2 mg/dL; specificity: 76.3%; sensitivity: 77.6%) reported as a potent biomarker of MAFLD in these patients [54]. Elsewhere, in a 10-year follow-up in Japan that recruited 17,021 participants (mean age: 49), Mori et al. found elevated levels of systolic blood pressure (SBP) over time in patients with MAFLD, compared to patients with fatty liver and patients “with FL but without MAFLD” [87]. Thus, when it comes to hypertensive patients, the “low-carb diet” therapy plan must be multi-functionalized with other modalities, such as (i) dispensing antihypertensive, glucose-lowering, and lipid-lowering drugs (to avoid hypertension, arterial stiffness, atherosclerosis, and coronary artery disease), (ii) prescribing antioxidants (to avoid inflammation and oxidative stress), and (iii) encouraging weight loss (to block the path by which obesity triggers CVD risk factors such as “diastolic and systolic dysfunction” and “cardiac arrhythmia and remodeling”) [88].

MAFLD is also interrelated with some manifestations of aging such as sarcopenia. For example, research shows that US individuals with sarcopenia (both young and middle-aged groups) are more prone to MAFLD (12.75%) than those without it (3.73%) [89]. Elsewhere, Zhou et al. reported that individuals with lower appendicular skeletal muscle mass (ASM)/BMI and ASM/weight (ASM/W) values are more predisposed to MAFLD, and MAFLD patients with lower ASM/W are at higher risk for HIR [90]. These and other findings emphasize that “age” is a critical aspect to consider, as age-related factors are directly or indirectly linked with this disease. Thus, the therapy plan needs to fix issues with lean body mass (LBM) which may appear in older groups. For example, a study of elderly Chinese subjects over 65 years of age showed that adequate intake of amino acids such as threonine (T; 750 mg/d), valine (V; 950 mg/d), and lysine (K; 1700 mg/d) (mostly found in foods like deep-sea fish, eggs, and milk) may mitigate the risk for NAFLD by up to 20%, while consuming 1000 mg/d aromatic amino acids (AAAs) may result in a 16% decline in this disease [91]. Similarly, a diet rich (in a controlled manner) with protein can aid older individuals with sarcopenia, as these individuals are fairly powerless to avoid inactivity (a major contributor to sarcopenia) [92]. Consequently, for this group of patients, treatment with a simple “low-carb diet” appears to be inadequate (or even the so-called “flog a dead horse”), and it requires a multifaceted undertaking of multiple factors.

##### Diet Low in FAs

FAs also play a role in the development of HS, and the dietary composition and pattern of FAs can determine the risk of the disease. Thus, low-fat diets are one of the primary therapies for HS. For example, research in 27 overweight Serbian men (27–42 years; BMI: 35 kg/m^2^) revealed that a DI with low FAs for 3 months could significantly reduce body weight (by >9%) and improve body fat (%), WC, VAI index, HS index [93], and fatty liver. It further reduced levels of TG, LDL cholesterol, total cholesterol, hepatic enzymes, insulin, and HOMA-IR index. However, MD exhibited better results (than a low-fat diet) in increasing levels of HDL cholesterol, monounsaturated FAs (MUFAs), and n-3 FAs (e.g., docosahexaenoic acid (DHA)) [94]. Research further indicates the efficacy of other relevant interventions, such as herbal regimes like a Chinese ECD diet (Erchen decoction, made up of six herbal items) to alleviate hepatic metabolic disorders and steatosis (in vivo in rats) by overproduction of butyric acid and promoting β-oxidation of fatty acid [95], or the dietary supplementation (5%) of fiber “butyrate” to attenuate this disease by positively affecting lipid metabolism in hepatocytes in wild-type (WT) mice in vivo [96]. Table 3 summarizes some of the recent findings regarding the consumption of low FAs for the treatment of HS.

**Figure 5:**
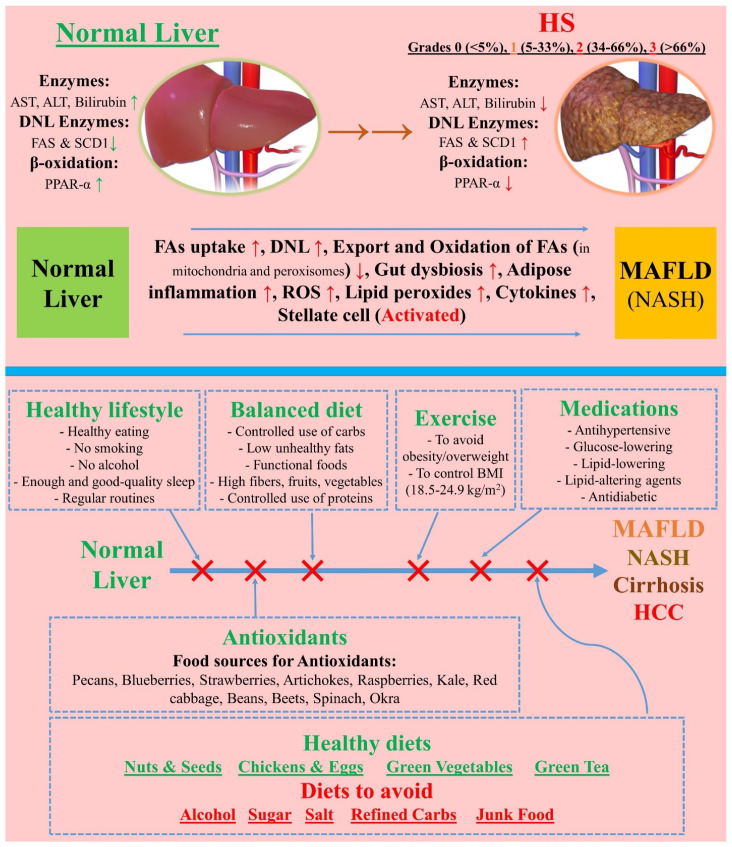
Mechanisms and enzymes involved in the development of HS, and therapy options which can avoid MAFLD (and subsequent NASH, cirrhosis, and HCC). Note that the content in this figure is only suggestions and needs to be approved in clinical trials [103]. HS: hepatic steatosis; MAFLD: metabolic dysfunction–associated FLD; NASH: non–alcoholic steatotic hepatis; AST: aspartate transaminase alanine; ALT: alanine transaminase; DNL: de novo lipogenesis; FAS: fatty acid synthase; SCD1: stearoyl–CoA desaturase; PPAR–α: peroxisome proliferator-activated receptor–α; FAs: fatty acids; ROS: reactive oxygen species; BMI: body mass index; HCC: hepatocellular carcinoma. This content is distributed under the terms of the Creative Commons Attribution 4.0 International license.

However, the interplay between the low content of FAs and other factors such as carbohydrates and proteins need to be reflected to elucidate their combined effect on the metabolic health of the liver. For example, Properzi et al. reported that both “low-fat” and “Mediterranean” diets can (to a similar degree; i.e., from 25% to 35%) attenuate HS, as evidenced in an RCT enrolling 48 patients with HS for 12 weeks. They found significantly higher total FAs and MUFAs (but low sodium and carbohydrates) in MD patients, compared to those on a low-fat diet, regardless of weight loss, suggesting the “low-fat + Mediterranean” diet as a preferred choice over Western foods [101]. Elsewhere, Gepner et al. showed that the “Mediterranean + low-carb” (MED/LC) diet is more effective than the “low-fat” diet in alleviating HS. They studied 278 participants for 18 months in a weight-loss trial and found that MED/LC outperforms the “low-fat” diet in reducing HFC (by %) and improving cardiometabolic risk parameters [102]. These results imply that, instead of pursuing a single therapy plan (such as weight loss alone or only consuming low-fat diets), it is more rational to devise multifaceted therapy plans that comprise multiple factors to cover all the contributors to HS incidence and development. For example, since inflammation of adipose tissue and adipokine dysbiosis critically contribute to HS and then NASH, cirrhosis, and even HCC (by activating stellate cells by recruiting “ROSs, lipid peroxides, cytokines, macrophages (TGF-β, CCL3, CCL5, IL-1β, and MCP1) and kupffer cells”), Figure 5 illustrates some tactics to avoid these conditions [103]. Nonetheless, the content in this figure is general and supposed to be beneficial for overall HS, and the therapy plan for HS should be tailored to each individual with specific conditions.

#### 2.3.2. Dietary Components for MAFLD

**Figure 6:**
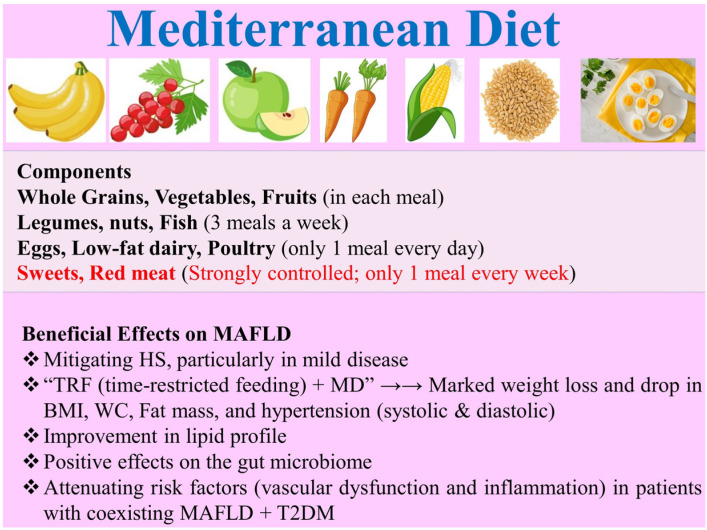
The Mediterranean diet (MD), its food components, and benefits for MAFLD patients. MD: Mediterranean diet; TRF: time-restricted feeding; BMI: body mass index; WC: waist circumference; T2DM: type 2 diabetes mellitus; MAFLD: metabolic dysfunction-associated fatty liver disease.

Overall, dietary components play a pivotal role in the prevention or development of HS. Recently, weight loss programs have become popular, particularly among those with hepatic disease. However, these programs require being complemented with a tailored and balanced diet that (i) aids the energy-burning workout to achieve measurable results and (ii) supplies the body with all the essential nutrients needed for optimal metabolism. For example, adopting an “isocaloric diet” plus adequate physical exercise is supposed to be effective in alleviating hepatic inflammation in patients with NAFLD by modulating inflammatory cytokines and adipokines [104]. Similarly, diet acceptance by the target population is also strongly in play. In a study of the Australian population with NAFLD, for example, patients preferred MD (rather than a low-fat diet) primarily due to its better flavor and enjoyment of food, novelty and easy swapping [105]. Most importantly, the dietary pattern must best fit each patient with a particular associated metabolic disorder(s). For example, a recent study investigated the efficacy of “dapagliflozin” (10 mg), “OM-3CA” (4 g), or merged “dapagliflozin + OM-3CA” in 84 Swedish patients (mean age: 65.5 years) with coexisting “NAFLD + T2DM”. It was found that hepatic proton density fat fraction (PDFF) was reduced by −21% when adopting the merged “dapagliflozin + OM-3CA”, compared to the corresponding values of 15% (OM-3CA) and 13% (dapagliflozin). Dapagliflozin was found to promote glucose control and mitigate hepatocyte injury biomarkers, suggesting its potential for use in dietary plans for patients with “NAFLD + T2DM” [106]. Consequently, dietary components can largely determine the success of nutritional interventions in the therapy of HS. For example, of all DIs, MD is well established and has many benefits to alleviate HS (Figure 6). Research strongly prefers MD (as a gold benchmark to treat MAFLD) over the Western diet [107]. This is mainly due to the capacity of MD to reduce body weight, BMI, hip circumference, WC, fat mass, and hypertension (both diastolic and systolic) [108]. “MD” plus “intermittent fasting” has been reported to be highly beneficial for MAFLD patients [109]. MD has also been reported to positively affect the gut microbiome, with a marked proliferation in the Bacteroides population of *Bacteroides* (effective in protecting against pathogens and carrying nutrients to other gut microorganisms) and *Paraprevotella* (effective in maintaining intestinal homeostasis), thus adjusting metabolic pathways in patients with MAFLD [110].

Apart from the type of diet, any DI should be administered in parallel with lifestyle adjustments, adequate physical activity, and abundant nutrients. For example, with regard to sufficient nutrients, research shows that including fruits in the diet will improve lipid metabolism, modulate insulin signaling pathways, block the enzymatic action of histone acetyltransferases, and mitigate liver inflammation [17]. For physical activity, research implies that the risks of “ACM” and “death from cancer” in NAFLD patients are reduced by 56% in patients pursuing regular leisure time physical activity (LTPA) [111]. Elsewhere, physical activity has been reported to reduce the incidence of sarcopenia, fibrosis, and CVD in NAFLD patients [112], and improve body composition, cardiorespiratory fitness, and cardiovascular health in individuals [113].

Most importantly, when proposing a diet for MAFLD patients, the dietary components must be useful for associated comorbidities such as CVDs and T2DM and contributing risk factors. For example, CVDs are well known to be caused by oxidative stress, irregular lipoprotein metabolism, and chronic inflammation. The dietary program for patients with co-existing CVDs and MAFLD, therefore, can contain (1) adequate fiber (as it reduces blood pressure (BP), inflammation, TC, and LDL-C), (2) herbal, whey, and egg proteins (all reducing BP), (3) MUFAs and PUFAs (all reducing BP, TC, TG, LDL-C, and inflammation, while increasing HDL-C), (4) vitamins A and C (all reducing ROS production), (5) vitamin E (lowering ROS production, inflammatory mediators, thrombosis, concentrations of serum lipids), (6) carotenoids (decreasing ROS production, LDL-C, apoptosis, and TC), and (7) food sources such as walnuts (lessening TG, TC, LDL-C, BP, inflammation) [114]. For example, research indicates that a diet rich in (i) root vegetables and green leafy, cooked, and cruciferous vegetables/salad, (ii) nuts (but in a controlled manner; e.g., tree nuts, peanuts), and (iii) whole grains (e.g., cereals, oats and oatmeal) will mitigate the risk of all-cause mortality, whereas “red and processed meat” are intensely harmful for patients with CVDs [115]. Similarly, the Dietary Approaches to Stop Hypertension (DASH) diet can be a proper option for MAFLD patients with hypertension. DASH is based on consuming more magnesium, potassium, calcium, vegetables, healthy proteins, fruits, whole grains, and fibers, while strongly advising against the use of salt, sugars, and saturated fats [116]. Collectively, such findings are useful when scheduling a diet for patients with co-existing MAFLD and associated diseases such as CVDs and T2DM. Accordingly, similar research is worth exploring within clinical trials to devise a versatile DI for those suffering from HS.

## 3. Conclusions

Management of NAFLD/MAFLD/MASLD requires a holistic approach that includes lifestyle modifications, such as dietary changes, exercise, and weight loss. These interventions are effective in reducing liver fat content and promoting liver function. Regular follow-up and monitoring are crucial to assess disease progression and detect complications. Upon a healthy lifestyle and under a tailored healthcare regimen, individuals with NAFLD/MAFLD/MASLD can successfully manage their condition and alleviate the risk of long-term complications. As mentioned, a treatment plan that can address both MASLD and CVD risk factors is vital to ensure that patients receive the best possible treatment for their condition and achieve optimal health outcomes. After considering evidence from endpoint justifications in cardiovascular trials and reflecting all aspects of medications (i.e., benefits, side effects, and licensed indications), researchers can decide whether these drugs can synergistically attenuate CVD risks and alleviate MASLD. Most importantly, current therapeutic plans for this condition (pharmacological, DIs, etc.) come with some shortcomings, and it is currently a daunting task to find a multifunctional and versatile therapeutic plan covering all the disease aspects. Thus, the field needs to focus mostly on emerging evidence, particularly from clinical trials, to allow for better understanding the disease aspects (to be satisfactorily overcome) and illustrate the efficacy of therapeutic interventions among human populations. These can be a pivotal topic in future studies and can critically reveal the current standing of currently proposed therapy plans in the real “from bench-to-bedside” axis.

## Figures and Tables

**Figure 4:**
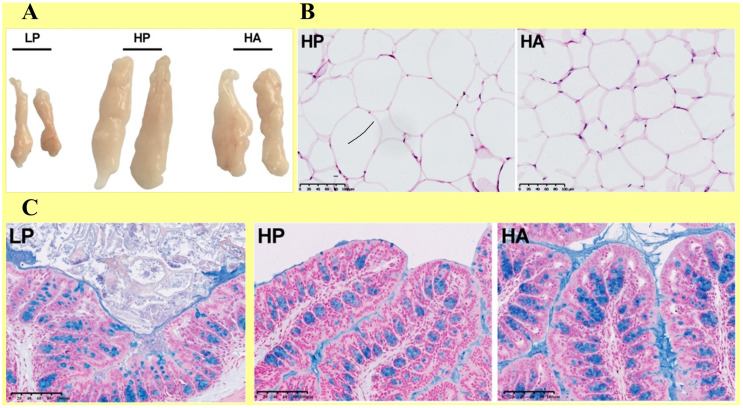
The HA diet (low fat + *A. muciniphila*) is supposed to alleviate HS, slow down weight loss, and relieve hepatic injury. It further mitigates “adiposity” and “alteration of adipokines”, both of which are contributors to MAFLD pathogenesis. Overall, the size of the total white adipose tissue (WAT) is reduced in HA-fed mice. Specifically, the size of the epididymal WAT tissue (eWAT) is reduced in the HA diet (compared to the HP diet = high fat) (**A**), while treatment with HA (compared to HP) reduces the size of adipocytes (**B**). Notably, one of the hallmarks of MAFLD is highly permeable intestinal barriers, which enhance the risk of systemic inflammation due to over transportation of bacteria (and bacterial products) and commensal metabolites. As shown in (**C**), the HA diet makes the “mucus layer and tight junctions” thicker (compared to the thinner layer in HP-fed mice). These suggest “*A. muciniphila*” as a potent probiotic that can bring beneficial outcomes and is worth exploring in future human clinical trials [100]. HA (low fat + *A. muciniphila*), HP (high fat), LP (low fat), WAT, eWAT. This content is distributed under the terms of the Creative Commons Attribution 4.0 International license.

**Table 1:** Recent findings suggestive of carbohydrates contributing to MAFLD occurrence and progression.

Research Background/Objectives and Stimuli for MAFLD	Target Group	Research Findings	Highlights (Effects on/and Contribution to MAFLD)	Ref.
To elucidate if overfeeding carbohydrates triggers de novo lipogenesis) DNL (and then HS)	Healthy young adults (*n*: 10) chosen for human experiments via direct infusion mass spectrometry (DI-MS)	A subset of triacylglycerols (TAGs) links with HS; in Western populations, HS is partly emanated from DNL after consuming a diet rich in carbohydrates	Compared to fasting, overfeeding carbs raises blood plasma levels of triacylglycerols (TAGs), with a change towards the formation of TAGs with shorter chains and fewer double bonds, suggesting a DNL-mediated conversion of carbs to TAGs, thus increasing the risk of HS.	[60]
To elucidate “fats-carbs” interaction and its contribution to intrahepatic triglyceride (IHTG) content	Evidence from humans; using data from stable isotope studies	Both glucose and fructose enhance content of IHTG	(A) Low-carbs-high-fat diets (with an excessive content of SFAs—saturated fatty acids) contribute more to an elevated IHTG content than high-carbs-low-fat diets. Refined carbs and free sugars enhance the risk of NAFLD. (B) SFAs and fructose exhibit the greatest contribution to elevated IHTG content Low intake of SFAs and strictly controlled use of added sugars (e.g., sugary beverages like soft sugared coffee and tea, soft drinks) can alleviate the content of IHTG	[61,62]
To explore the effects of various types of carbs on the occurrence of HS	Patients with NAFLD (*n*: 660; aged 35–70) (Data from Iran)	NAFLD is positively associated with total dietary carbs, i.e., higher content of carbs (regardless of their type) and links with worsened state of NAFLD	Compared to healthy individuals, patients with NAFLD consume more fructose and glucose.	[63]
To identify the effects of fructose, glucose, and fibers (as dietary carbs) and the gut microbiota on the occurrence of HS	Evidence from humans	Diets rich in carbs and mechanisms driven by microbial dysbiosis/metabolism trigger hepatic lipogenesis, thereby NAFLD	Metabolism of carbs can trigger and feed DNL, as diets rich in carbs elevate hepatic lipogenesis. Soluble fibers (e.g., insulin, pectin, β-glucan, etc.) produce short-chain FAs (SCFAs), and SCFAs are beneficial (e.g., anti-DM, anti-obesity, and anti-tumor effects). However, excess intake of soluble fibers may trigger hepatic lipogenesis (by delivering more acetate that feeds DNL.	[64]
To compare effects of low-carb-high-fat (LCHF) diet vs. high-carb-low-fat (HCLF) diet on the occurrence of T2DM and HS	T2DM patients (*n*:165; mean age: 56; 58% were females) (Data from Denmark from 6-month RCT and 3-month follow-up)	Glycemic control and weight loss are more visible in patients driving with LCHF (with unrestricted calories), compared to those inclined to HCLF. LCHF diet results in low LDL cholesterol content. NAFLD activity score (NAS) was improved by 2 or more points in 16 patients (on the LCHF diet) and 6 patients (on the HCLF diet), implying the efficacy of the LCHF diet in alleviating HS.		[65]
Comparison of HS occurrence among children and adults	Evidence from the literature	The clinical and economic burden of HS is increasing globally. This condition adversely affects the quality of life in both groups.	NAFLD prevalence is 8 to 12% (among children) and 25 to 48% (among adults); in affected patients, NASH prevalence is 23% (among affected children) and 13 to 65% (among affected adults). These findings suggest a higher incidence of the disease with aging.	[66]

Abbreviations: MAFLD: metabolic dysfunction-associated FLD; TAGs: triacylglycerols; DNL: de novo lipogenesis; HS: hepatic steatosis; IHTG: intrahepatic triglyceride; SFAs: saturated fatty acids; SCFAs: short-chain fatty acids; DM: diabetes mellitus; LCHF: low-carb-high-fat; HCLF (high-carb-low-fat; T2DM: type 2 diabetes mellitus; RCT: randomized controlled trial; NAS: NAFLD activity score; LDL: low-density lipoprotein).

**Table 2:** Effects of diets low in carbohydrates on reducing MAFLD occurrence and progression.

Study Protocol/Objectives and Intervention	Target Group	Findings	Highlights	Ref.
To explore the effects of merged “low-carb diet” plus “TRF (time-restricted feeding)” on HS	NAFLD patients attended an RCT for 84 days; Therapy plan: “low-carb-diet” + “TRF (8 h feeding/16 h fasting) (TRF 8/16)” (Data from Iran)	Diet low in sugar plus TRF is effective in reducing the occurrence of NAFLD	A significant decline in levels of adiposity (e.g., by decreasing BMI, body weight, body fat, WC) and an improvement in liver, inflammatory, and lipid markers. The rate of NAFLD did not decline, but the therapy plan is beneficial to patients by alleviating associated discomfort.	[67]
Comparing “a low-carb diet” with “a low-fat diet” and “MD”	Data from RCTs on human populations	Both MD and low-carb diets can potentially reduce hepatic fat content	In patients with NAFLD (but without T2DM and NASH), the hepatic fat content is much more reduced when adopting MD (compared to two other treatments) When excluding T2DM, MD comes with more noticeable benefits (in reducing TG and hepatic fat) than the two other treatments	[68]
Lipid metabolism modulation by low consumption of carbohydrates (to explore the hepatoprotective effects of low-fat diets)	Evidence from the literature on human populations	Carb restriction improves NAFLD	Adopting a ketosis diet (i.e., using <30 g/day carbs) improves NAFLD by inducing ketogenesis	[69]
Effects of low-free sugar diets on NAFLD	43 overweight/obese patients with NAFLD	A diet low in free sugars markedly decreases levels of ALT, TG, TC, FBS, insulin, HOMA-IR, TNF-α, and NF-kb Alleviated HS and fibrosis, improved glycemic indices, and decreased levels of inflammation biomarkers		[70]
RCT study of effects of intermittent fasting and low-carb high-fat (LCHF) diet on HS	74 NAFLD patients (data from Sweden)	Both intermittent fasting (the 5:2 diet) and LCHF exert equal effects on decreasing HS and body weight The 5:2 diet outperforms the LCHF diet in reducing LDL cholesterol and liver stiffness The 5:2 diet is more tailored to NAFLD patients (particularly those with CDVs)		[71]

Abbreviations: MAFLD: metabolic dysfunction-associated FLD; TRF: time-restricted feeding; BMI: body mass index; WC: waist circumference; MD: Mediterranean diet; TG: triglyceride; NASH: nonalcoholic steatohepatitis; ALT: alanine transaminase; TC: total cholesterol; FBS: fasting blood glucose; HOMA-IR: homeostasis model assessment of insulin resistance; TNF-α: tumor necrosis factor–α; NF-kb: nuclear factor kappa B; HS: hepatic steatosis; CDVs: cardiovascular diseases.

**Table 3:** Therapeutic strategies and solutions proposed and explored to treat HS regarding the consumption of FAs (recent findings).

Study Protocol/ Intervention	Target Group	Highlights	Ref.
Assess different FA patterns in the treatment of HS	Participants from the UKB (age: 40 to 69)	A vegetarian diet rich in PUFAs (containing DHA (docosahexaenoic acids) and LA (Linoleic acid)) can alleviate simple HS, while a carnivore diet rich in PUFAs does not affect the disease (progression or control).	[97]
Explore the impacts of ω3 PUFAs on HS (regarding that low levels of C_20_–_22_ ω3 PUFAs contribute to disease progression)	Evidence from literature	A diet rich in C_20_–_22_ ω3 PUFA helps alleviate NAFLD severity, as it reduces hepatic injury and hepatosteatosis Tips: “Dietary supplementation with C_20_–_22_ ω3 PUFAs” alone will not achieve measurable results and needs to be in tandem with pharmaceutical therapies. DHA outperforms EPA (eicosapentaenoic acid) in delaying the onset and progression of NAFLD.	[98]
The usefulness of an MD rich in unsaturated FAs (UFAs) (with or without pentadecanoic acid (C15:0))	An RCT targeting Chinese female patients with NAFLD (*n*: 88)	The highest weight loss (~4 kg) is obtained by “diet with C14:0”, followed by “diet without C14:0” (~3.4 kg) and controls (normal diet without C14:0; 1.5 kg) (with, respectively, 33%, 30%, and 10% reduction in hepatic proton density fat fraction (PDFF) values Dietary intervention (compared to control) reduces hepatic PDFF, body weight, total cholesterol, gamma-glutamyl transferase (GGT) enzyme, and TG Dietary supplementation with C15:0 outperforms the diet without C15:0 in reducing levels of LDL cholesterol An Asian diet rich in PUFA, MUFAs, and fiber is helpful for weight reduction and improving hepatic health and metabolic profile.	[99]
Comparing the effects of various fat diets: (1) “HP (high fat)”, (2) “LP (low fat)”, and (3) “low fat with *Akkermansia muciniphila* (HA)” on HS in C57BL/6 mice in vivo	C57BL/6 mice (6-W old)	HA (low fat + *A. muciniphila*) mitigates HS, weight gain, and hepatic injury HA improves glucose tolerance, dysbiosis of adipokines There is a nexus between metabolic disorders, microflora, and probiotics (Figure 4) *A. muciniphila* can be a potent probiotic therapy for HS	[100]

Abbreviations: HS, hepatic steatosis; FA, fatty acid, UKB, United Kingdom Biobank, PUFAs, polyunsaturated fatty acids, DHA, docosahexaenoic acids, LA, linoleic acid, EPA, eicosapentaenoic acid, PDFF, proton density fat fraction, GGT, gamma-glutamyl transferase, MUFAs, monounsaturated fatty acids, HA, low fat + *A. muciniphila*, HP, high fat, LP, low fat.
